# Development of Scaffolds with Chitosan Magnetically Activated with Cobalt Nanoferrite: A Study on Physical-Chemical, Mechanical, Cytotoxic and Antimicrobial Behavior

**DOI:** 10.3390/ph17101332

**Published:** 2024-10-05

**Authors:** Danyelle Garcia Guedes, Gabryella Garcia Guedes, Jessé de Oliveira da Silva, Adriano Lima da Silva, Carlos Bruno Barreto Luna, Bolívar Ponciano Goulart de Lima Damasceno, Ana Cristina Figueiredo de Melo Costa

**Affiliations:** 1 Laboratory of Ceramic Materials Synthesis, Federal University of Campina Grande, 882 Aprígio Veloso Street—Bodocongó, Campina Grande 58429-900, PB, Brazil; 2 Pharmaceutical Product Development and Characterisation Laboratory, State University of Paraíba, 351 Baraúnas Street—Universitário District, Campina Grande 58429-500, PB, Brazil; 3 Drug Development and Testing Laboratory, State University of Paraíba, 351 Baraúnas Street—University District, Campina Grande 58429-500, PB, Brazil; 4 Materials Engineering Academic Unit, Polymer Processing Laboratory, Federal University of Campina Grande, Av. Aprígio Veloso, 882, Campina Grande 58429-900, PB, Brazil

**Keywords:** biomaterials, freeze-drying, antimicrobial properties, magnetism, pilot scale

## Abstract

**Background/Objectives**: This study investigates the development of 3D chitosan-x-cobalt ferrite scaffolds (x = 5, 7.5, and 10 wt%) with interconnected porosity for potential biomedical applications. The objective was to evaluate the effects of magnetic particle incorporation on the scaffolds’ structural, mechanical, magnetic, and biological properties, specifically focusing on their biocompatibility and antimicrobial performance. **Methods**: Scaffolds were synthesized using freeze-drying, while cobalt ferrite nanoparticles were produced via a pilot-scale combustion reaction. The scaffolds were characterized for their physical and chemical properties, including porosity, swelling, and mechanical strength. Hydrophilicity was assessed through contact angle measurements. Antimicrobial efficacy was evaluated using time kill kinetics and agar diffusion assays, and biocompatibility was confirmed through cytotoxicity tests. **Results**: The incorporation of cobalt ferrite increased magnetic responsiveness, altered porosity profiles, and influenced swelling, biodegradation, and compressive strength, with a maximum value of 87 kPa at 7.5 wt% ferrite content. The scaffolds maintained non-toxicity and demonstrated bactericidal activity. The optimal concentration for achieving a balance between structural integrity and biological performance was found at 7.5 wt% cobalt ferrite. **Conclusions**: These findings suggest that magnetic chitosan-cobalt ferrite scaffolds possess significant potential for use in biomedical applications, including tissue regeneration and advanced healing therapies. The incorporation of magnetic properties enhances both the structural and biological functionalities, presenting promising opportunities for innovative therapeutic approaches in reconstructive procedures.

## 1. Introduction

With the growth of tissue engineering (TE), the strategic design of scaffolds has become essential for the regeneration and repair of various tissue types, including bone [1], cartilaginous [2], muscular [3], and nervous tissues [4]. In biomaterials, the term “scaffold” refers to three-dimensional structures designed to provide physical and biological support for the growth and regeneration of tissues and organs [5]. These 3D structures are designed to (i) mimic the extracellular matrix (ECM), creating a more natural environment that supports cell adhesion, migration, differentiation, and proliferation; (ii) provide adequate mechanical support; (iii) guide regeneration; (iv) enable a controlled release of bioactive agents; and (v) minimize inflammatory responses, aiming at the formation of functional and integrated tissue [6,7]. In light of this, developing biological scaffolds with optimized properties requires a careful selection of materials and manufacturing methods. Customizing this technology by incorporating advanced functionalities, such as responsiveness to magnetic fields, not only enhances versatility but also expands the possibilities for targeted manipulation of cellular processes, highlighting the innovative nature of these new devices for applications in tissue engineering [8].

Recent trends in tissue engineering have guided research toward the development of magnetically responsive scaffolds [9]. With this purpose, magnetic nanoparticles (MNPs) are incorporated into biomaterials to influence tissue growth due to their responsiveness to externally applied magnetic fields [10]. MNPs induce a mechanical response that stimulates the differentiation of stem cells into the cell types needed for tissue regeneration, facilitated by the release of growth factors that promote the mineralization of the extracellular matrix [11]. The magnetic force also improves cell migration to the injury site, accelerating healing and scaffold integration [10]. In addition, MNPs release angiogenic growth factors that favor the formation of new blood vessels, which are essential for supplying nutrients and oxygen to regenerative cells [11]. The ability of magnetic scaffolds to release bioactive molecules in a controlled manner makes it possible to direct the cellular response, enhancing treatment and tissue regeneration [10]. This strategy paves the way for the controlled release of drugs [12], proteins, and cells [13,14], enabling non-invasive therapeutic intervention after scaffold implantation [15]. Additionally, MNPs modify the mechanical properties of the scaffold, making it more resistant and stable, which is crucial for effective cell growth support and tissue regeneration. Despite these advantages, the safe use of magnetic scaffolds in tissue engineering still requires further research to ensure the viability of the materials used.

Given this challenging scenario, chitosan (CS) emerges as a high-potential organic matrix for creating magnetic scaffolds. Chitosan, a natural polymer derived from chitin, is known for its biocompatible, biodegradable, antibacterial, antifungal, and hemostatic properties [16,17]. Incorporating MNPs into chitosan scaffolds preserves biological compatibility, ensuring that the scaffold is safe for use in biomedical applications. The chitosan matrix stabilizes MNPs and ensures their controlled release as the scaffold degrades, safely eliminating them without causing toxicity. Its biocompatible surface limits protein adsorption and modulates cellular uptake, affecting key processes like opsonization and endocytosis. In this context, macrophages—critical effectors of the foreign body response—recognize opsonized MNPs marked by proteins and internalize them through endocytosis [11,18]. This leads to the clearance of MNPs, with the liver and spleen showing the highest uptake. As the scaffold dissolves, chitosan minimizes adverse effects by releasing MNPs gradually [19]. Recent studies have reported the successful fabrication of magnetic scaffolds. Babakhani et al. [20] developed scaffolds based on polyvinyl alcohol and chitosan, incorporating hydroxyapatite (HAp) and clay modified with graphene oxide and magnetite (Fe_3_O_4_) nanoparticles. The modified clay enhanced the compressive strength, improved the scaffold’s surface mineralization, and demonstrated good cell viability. Azadani et al. [21] incorporated magnetic mesoporous bioactive glass (MMBG) into polyhydroxybutyrate (PHB) coated with chitosan and carbon nanotubes. It was observed that these scaffolds promoted macrophage polarization, shifting their activity from pro-inflammatory to anti-inflammatory and repair functions.

Magnetic scaffolds can be manufactured by incorporating iron oxide nanoparticles into polymeric matrices. These nanoparticles naturally occur in three forms: hematite (α-Fe_2_O_3_), maghemite (γ-Fe_2_O_3_), and magnetite (Fe_3_O_4_) [22]. These materials offer advantages such as biocompatibility, low toxicity to the human body, oxidation resistance, and a stable magnetic response over the long term, which can last up to 50 years [23]. For effective use in biomedical applications, such as controlled drug delivery, MNPS must have high saturation magnetization (Ms), along with low coercive field (Hc) and remanent magnetization (Mr) values [24]. Magnetite is the most commonly used in biomedical applications due to its high saturation magnetization (Ms). However, its tendency to oxidize into maghemite in biological environments can compromise its magnetic properties over time, potentially reducing its effectiveness in applications such as targeted drug delivery or magnetic hyperthermia [25].

In this context, cobalt ferrite (CoFe_2_O_4_, CFO) emerges as a unique strategy for the development of biomedical scaffolds. Its chemical stability, mechanical hardness, electrical insulation, wear resistance, non-toxicity, and ease of synthesis collectively provide a promising combination of properties [26,27]. The combustion reaction stands out as a promising synthesis route among the various techniques available. This approach allows for the rapid and homogeneous formation of CFO in a single step, resulting in uniform particles and providing precise control over their properties. Additionally, the CFO nanoparticles produced by this method exhibit effective dispersion, promoting better integration with the scaffold matrix, compatibility with host tissue, and increased cell viability [28,29,30]. Furthermore, Co²⁺ ions in the CFO structure have therapeutic effects and antibacterial activity, making it an intriguing alternative for tissue engineering (TE) and other biomedical applications [31,32,33,34].

To advance scientific and technological research in the interdisciplinary fields of materials science and engineering and tissue engineering, this study proposes the development of chitosan scaffolds magnetically activated with cobalt ferrite produced by a combustion reaction on a pilot scale. A recent literature review indicates that this material has yet to be used in synthesizing tissue scaffolds [35]. Therefore, we investigated the impact of incorporating 5, 7.5, and 10% cobalt ferrite on the physical, mechanical, and biological properties of the scaffolds, with a focus on their potential for applications in tissue engineering.

## 2. Results and Discussion

### 2.1. Physicochemical Results

**Figure 1:**
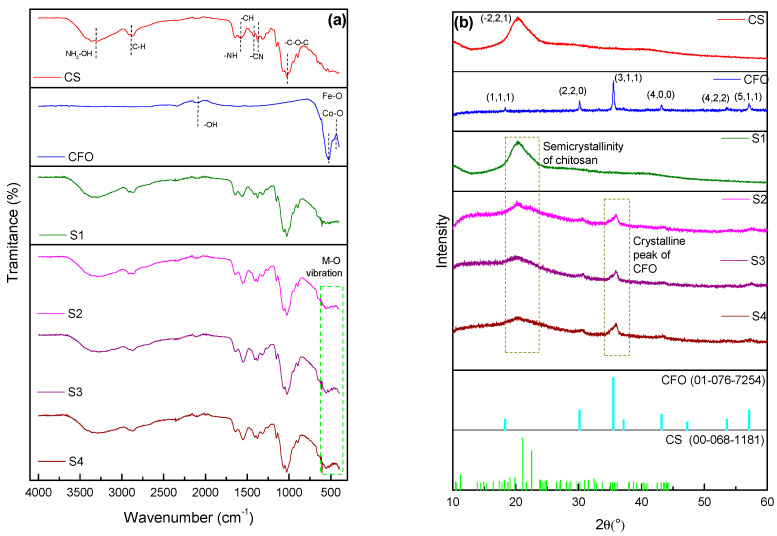
(**a**) FT-IR spectra and (**b**) XRD patterns of chitosan-CS, cobalt ferrite-CFO, reference specimen-S1, and magnetic scaffolds S2, S3, and S4.

The FT-IR spectra and X-ray diffraction patterns of the structures produced by lyophilization are shown in Figure 1a,b.

In Figure 1a, for scaffolds S2, S3, and S4, the analysis revealed the presence of multiple bands representing functional groups, thus confirming the successful synthesis of chitosan polymeric structures. Table 1 compares the characteristic vibrations observed in the chitosan and cobalt ferrite scaffolds with the literature data, as identified by the FT-IR spectra.

The absorption band observed at 451 cm^−1^ and the weak vibration at 562 cm^−1^ confirm the metal–oxygen (M–O) stretching vibrations of Fe^3+^-O^2−^ or Co^2+^-O^2−^ in the spinel cobalt ferrite at site (A) and site [B], respectively. Similar behavior has been previously reported by Anila et al. [36]. Additionally, the bands observed at frequencies of 888, 1021, 1367, and 1570 cm^−1^ can be attributed to glycosidic bond stretching, C–O stretching, symmetric C–O–C stretching, C–N stretching, O=C–CH stretching of the amide, N–H bending associated with the primary amide group resulting from the deacetylation process, and C=O stretching (amide I), as well as the amino group. Similar behavior has been previously reported by Han et al. and Zhang et al. [37,38] when researching biomaterials containing chitosan. Finally, the pronounced and broad bands located approximately at 3356 and 3286 cm^−1^ in the spectra indicate characteristic absorption bands associated with the overlapping vibrations of O-H and N-H, which is consistent with the results obtained by Zhang et al. [38].

Figure 1b illustrates the diffraction pattern for the cobalt ferrite synthesized by the combustion reaction on a pilot scale, showing the inverted spinel structure as the major phase—according to the crystal pattern in the JCPDS 01-076-7254 database—with 66% crystallinity and a crystallite size of 31 nm. The peaks observed in the scaffolds S1, S2, S3, and S4 at 2θ = 10.4° and 20.2° indicate the characteristic semi-crystallinity band of chitosan. In the scaffolds containing 5, 7.5, and 10% CFO (S2, S3, and S4), there are four low-intensity peaks characteristic of the inverted spinel structure at the 2θ positions of 30.1°, 35.5°, 43.1°, and 56.9°. These peaks correspond to the crystalline planes (2 2 0), (3 1 1), (4 0 0), and (5 1 1), respectively, of the inverted spinel phase of CFO, which is consistent with the literature [29,36,39,40] In relation to studies involving cobalt ferrite, the clarity and intensity of these peaks indicate a well-defined crystallinity, validating the success of the synthesis of the magnetic supports.

**Figure 2:**
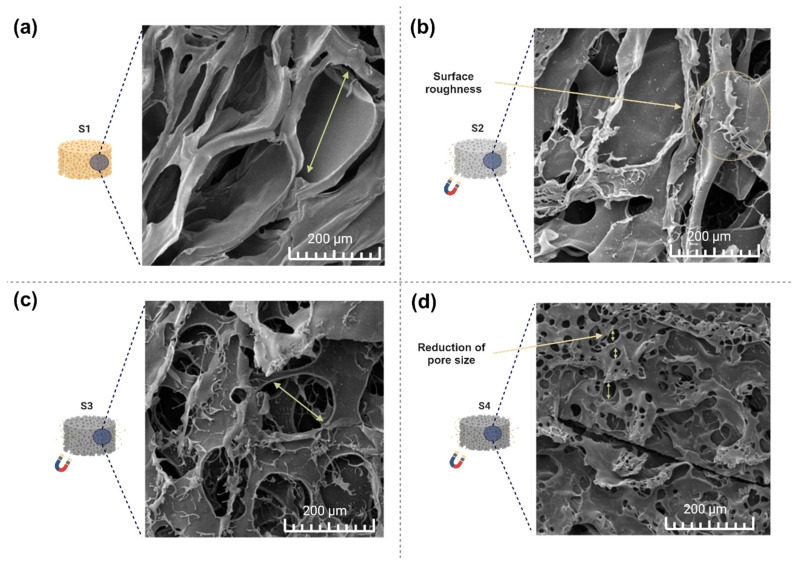
(**a**) SEM micrographs of a reference specimen, (**b**) S2, (**c**) S3, and (**d**) S4 magnetic scaffolds.

The morphology of the scaffolds was analyzed using scanning electron microscopy (SEM), as illustrated in Figure 2a–d.

As illustrated in Figure 2a, the reference scaffold exhibits a polymeric structure with uniform and smooth characteristics. Figure 2b–d indicates good dispersion of CFO within the polymeric matrix. The addition of a higher volume of magnetic material had a decisive impact on the scaffold morphology, resulting in a more compact and heterogeneous structure. Additionally, the increase in CFO percentage gradually rounded and reduced the pore size, promoting a more uniform pore organization. A similar behavior was observed by Tavares et al. [41] during their study on the optimization of magnetic chitosan (CS) and poly(vinyl alcohol) (PVA) structures integrated with hydroxyapatite (HAp), and by Farzaneh et al. [42] in producing polyvinylpyrrolidone (PVP) and CFO structures.

**Figure 3:**
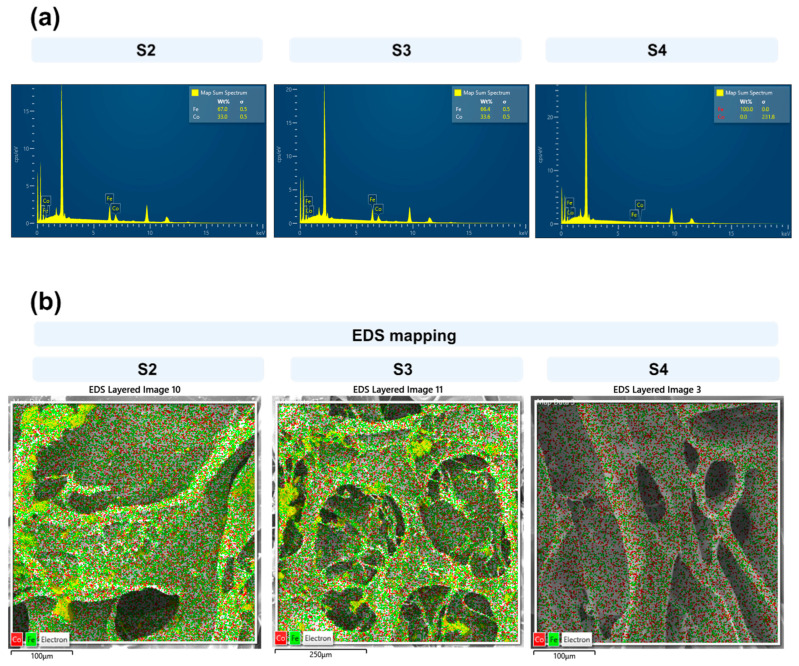
(**a**) EDS spectrum and (**b**) mapping images of three scaffolds showing the different distribution patterns of Fe and Co on the surface.

The chemical analyses by EDS are illustrated in Figure 3.

As observed in Figure 3a,b, the main metallic elements, including Fe and Co, were detected in the EDS mapping images derived from the magnetic particles within the scaffolds. The EDS mapping of Co and Fe indicates the incorporation of ferrite on the surface of the scaffold (Figure 3b).

Mercury porosimetry is described in Table 2.

The observed data indicate a significant variation in porosity and average pore diameter among the scaffolds. The incorporation of 5, 7.5, and 10% CFO into the scaffolds resulted in a notable change in the porosity of the structures. It was observed that the porosity increased to a range between 79% and 83%. According to Maia et al. [43], this is a desired range of porosity for effective simulation of trabecular bone. This modification in porosity is highly relevant, considering that characteristics such as the presence, size, and connectivity of pores are critical factors that directly influence cellular behavior, vascularization, and the ability of cells and nutrients to infiltrate the interior of the scaffold. In addition to the change in porosity, a significant variation in pore diameter was observed due to incorporating CFO. Specifically, in the reference scaffold (S1), a pore diameter of 104.9 μm was observed, whereas the addition of 5% CFO (S2) resulted in pores with an average diameter of 131.3 μm. However, subsequent increases in CFO concentrations led to a gradual reduction in pore diameter, with S3 (7.5% CFO) exhibiting a diameter of 120.5 μm and S4 (10% CFO) showing a diameter of 87 μm.

According to Yadac et al. [44], pores larger than 300 μm promote vascularization, while smaller pores, such as those with a diameter of 50 μm, are recommended for bone regeneration. Although the diameters observed in the study are below 300 μm, they still fall within a range that supports bone growth, especially when combined with pores of varying sizes. For instance, pores with diameters between 87 μm and 131 μm can facilitate cellular infiltration and capillarity, contributing to bone support and vascularization while also increasing cell diversity within the scaffold, as evidenced by the micropores. This demonstrates that the variation in porosity and diameter obtained is suitable for bone regeneration applications, balancing the benefits of different pore sizes.

This variation in pore size is of particular interest, as it affects cellular functionality and vascularization and may also have implications for the material’s mechanical strength, swelling properties, and degradation behavior [45]. The observed phenomenon highlights the importance of scaffold composition and processing in achieving optimized physical and biological properties for specific tissue engineering applications [46].

**Figure 4:**
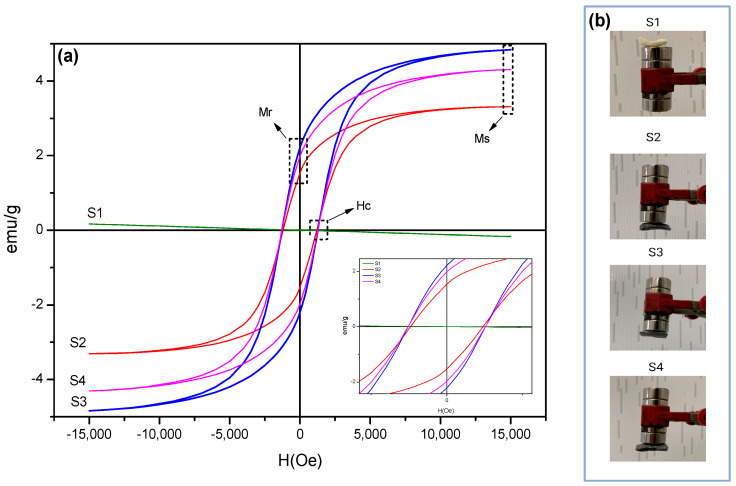
(**a**) Magnetization hysteresis loops and (**b**) digital photos of magnets interacting with various scaffolds.

Figure 4 illustrates the magnetization curves of the scaffolds S1, S2, S3, and S4.

Based on the hysteresis curves illustrated in Figure 4a,b, it is evident that the scaffolds exhibit ferrimagnetic behavior with a hard magnetic profile, consistent with the magnetic behaviors of the cobalt ferrites reported in the recent literature [27]. In contrast, S1 demonstrates the behavior of a diamagnetic material.

Table 3 details the results of the saturated magnetization (Ms), coercive field (Hc), and remanence (Mr) of the scaffolds.

Based on the data, it can be inferred that although the hysteresis curves are similar, the values of Ms and Mr vary. This variation can be attributed to the incorporation of magnetic particles into the chitosan polymer matrix and the specific structural profile resulting from scaffold processing. This structural profile is significantly influenced by the morphological characteristics induced by the freeze-drying process, such as the pore volume, surface roughness, and pore shape. While the ferromagnetic properties of the particles are preserved within the polymer scaffold matrix, the resulting morphological characteristics can have a considerable impact on the magnetic properties of the composites [47,48,49].

The saturation magnetization (Ms) values observed for scaffolds S2, S3, and S4 indicate that these materials can generate strong magnetic fields, facilitating the targeted accumulation of MNPs in specific tissues when subjected to an external magnetic field. This property is vital for applications where the precise localization of therapeutic agents is needed. The ability to control the magnetic properties via structural adjustments in the scaffolds can enhance the therapeutic outcomes while minimizing the off-target effects [19]. According to Vasić et al. [19], for applications such as targeted drug delivery, lower remanence and coercivity are often desirable to avoid unwanted agglomeration and ensure that the particles do not remain magnetized, which could lead to off-target effects. On that basis, the Mr and Hc observed values for magnetic scaffolds (S2, S3, and S4) indicate a favorable characteristic for ensuring that MNPs do not agglomerate, thus maintaining effective dispersion within the tissue and enhancing their biocompatibility.

**Figure 5:**
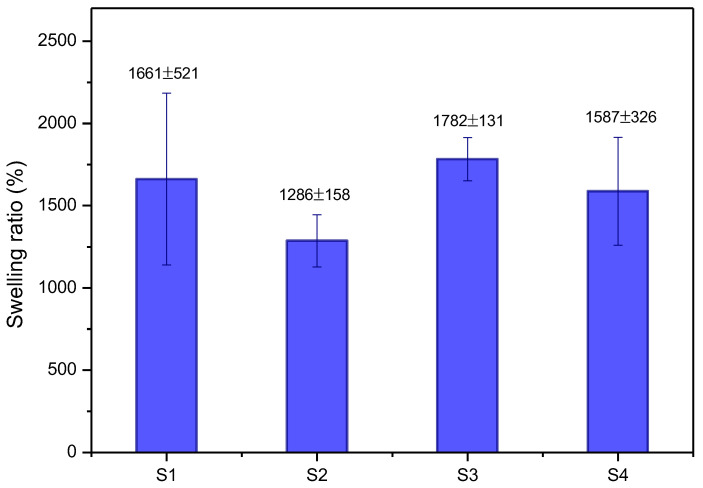
Swelling behavior of scaffolds in PBS. Error bars indicate mean ± SD (*n* = 5).

Figure 5 shows that the scaffolds’ swelling capacity was evaluated after immersion in a PBS solution (pH 7.3).

The swelling behavior of the scaffolds did not follow a linear trend with respect to the CFO concentration. The scaffold S3, with 7.5% CFO, exhibited the highest swelling index, reaching 1782%, whereas scaffolds S2 (5%) and S4 (10%) showed lower swelling capacities. This result suggests that structural hydration, polymer chain relaxation [50], and porous morphology [42] are crucial factors in the swelling process. For soft tissues, such as skin or cartilage, high swelling capacity can be beneficial, as it facilitates nutrient transport and hydration. However, for rigid tissues like bone, excessive swelling may compromise structural integrity, making it undesirable [51].

**Figure 6:**
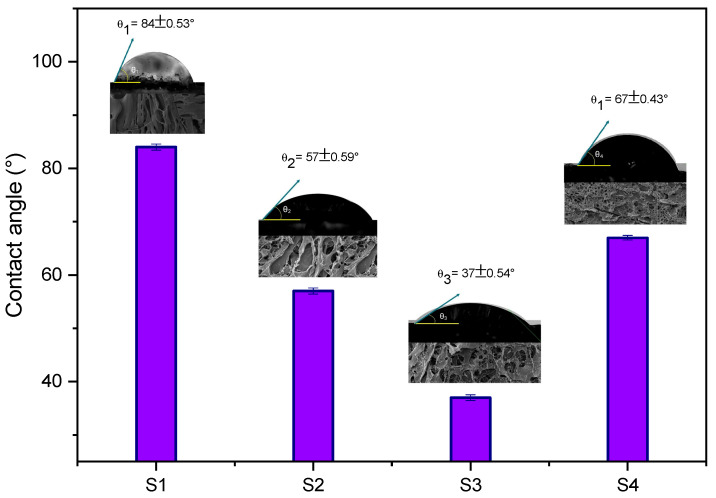
Contact angle of the S1, S2, S3, and S4 scaffolds. Error bars indicate mean ± SD (*n* = 5).

Figure 6 illustrates the interfacial behavior of the scaffolds with water.

Based on previous studies [23,52,53,54], it was anticipated that the concentration of CFO used in the fabrication of the scaffold would affect the mechanical properties, surface roughness, and hydrophilicity of the scaffolds. Hydrophilicity was determined using contact angle measurements. The contact angle of a surface with water is crucial for characterizing the material and indicates its absorption and adhesion properties. Surfaces with low contact angles are hydrophilic and hygroscopic, while highly wettable surfaces have contact angles below 20°. In contrast, hydrophobic surfaces have contact angles greater than 90° [55].

Figure 6 illustrates that the addition of CFO to CS led to a decrease in the contact angle. In comparison, for the reference scaffold (S1), the contact angle is 84°, while the contact angle of CS varies between 46° and 90° in various studies; this variation is observed based on the molecular weight and processing method. The decrease in contact angle demonstrates that CFO nanoparticles improved the hydrophilicity of the scaffold, with S3 being the most hydrophilic, with a CFO loading of 7.5% by weight.

The improved hydrophilicity was expected to increase the interactions between water molecules and the structures, as CFO is a highly hydrophilic material [55,56]. However, the contact angle increased with a CFO loading of 10 wt%. These results indicate that the wettability of the scaffold depends on factors such as its chemical composition, roughness, porosity, and crystallinity [21]. The hydrophilic behavior of porous structures is crucial for the efficient circulation of nutrients within the scaffold, playing a vital role in cellular processes, including adhesion, division, and proliferation [57,58].

**Figure 7:**
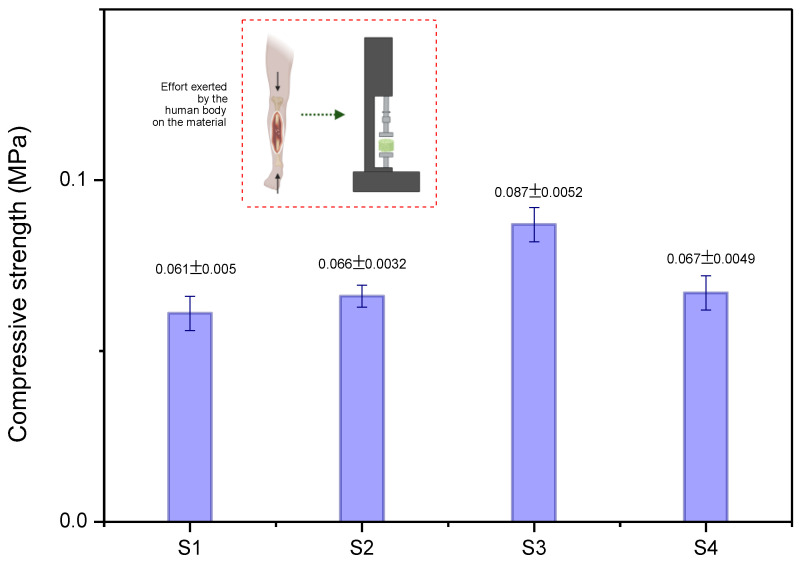
Mechanical strength values for scaffolds. Error bars indicate mean ± SD (*n* = 5).

Figure 7 illustrates the compressive strength of the scaffolds.

The data analysis in Figure 7 shows that the compressive strength of the scaffolds increased with the addition of CFO, attributed to the improved orientation of the polymer chains and electrostatic interactions between the ferrite nanoparticles and the polymer’s functional groups. This enhancement results in a higher density of cross-linking and, consequently, improved mechanical properties of the scaffold. Scaffold S3, with 7.5% CFO, exhibited the highest compressive strength, while S4, with 10% CFO, experienced a slight decrease in strength. This reduction is likely due to the formation of smaller pores, which act as stress concentrators, thereby impairing the mechanical properties of the scaffold—a phenomenon also observed by Farzaneh et al. [42].

For biomedical applications such as bone tissue engineering, scaffolds should have mechanical strengths ranging from 50 to 350,000 kPa [59]. The S3 scaffold demonstrated a compressive strength of 87 kPa, which falls well within the lower end of this required range. This suggests that the material is suitable for applications that require lower mechanical loads, such as in low-stress bone defects. Additionally, the combination of adequate porosity and balanced mechanical strength indicates that the S3 scaffold could provide a favorable environment for bone regeneration, with the potential for future optimizations to expand its use in various clinical contexts.

**Figure 8:**
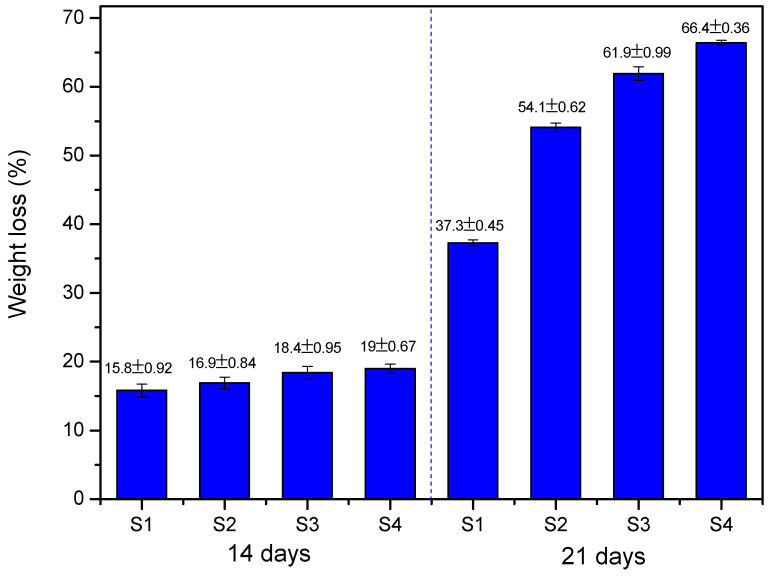
Degradation behavior of scaffolds after immersion in PBS solution, after 14 days and 21 days. Error bars indicate mean ± SD (*n* = 5).

Figure 8 illustrates the degradation behavior of the scaffolds over time.

The degradation results of the scaffolds over 14 and 21 days show a tendency for degradation to increase with immersion time in the PBS solution. After 14 days, the average degradation of the scaffolds ranged from 15.8% to 19%, with relatively low variability between the samples (S1 to S4). This initial degradation can be attributed to hydrolysis and initial exposure to the environment, where the material’s bonds begin to be broken, but without significantly compromising structural integrity.

The degradation rate is accelerated by the accumulation of water-soluble degradation products inside the polymer, which leads to the osmotic absorption of water by the matrix. These processes promote homogeneous mass erosion behavior, i.e., degradation occurs uniformly along the entire cross-section of the polymer matrix. It may be faster in the core of the matrix than on the outer surface. Mass degradation begins after a few days and is initially characterized by a reduction in molecular weight (without significant loss of mass for the scaffold structure) since the low molecular weight oligomers remain retained in the matrix. The release of these degradation products is accelerated as the erosion facilitates access to the polymer core [60].

After 21 days, the degradation values increased considerably, ranging from 37.3% to 66.4%. This substantial increase suggests an accelerated progression of degradation after the first 14 days, indicating that the scaffolds have entered a more advanced stage of deterioration, possibly due to the accumulation of structural damage over the immersion time. The loss of mass follows a trend proportional to the porosity of each sample. As the concentration of MNPs in the scaffolds increased, the mass loss became more pronounced.

As the structure of the magnetic scaffold degrades within the body, making room for the new natural tissue formed, the chitosan matrix is expected to stabilize the MNPs and ensure their controlled release. This release is conducted safely without causing toxicity since the biocompatible surface of chitosan reduces protein adsorption and modulates cellular uptake, impacting key processes such as opsonization and endocytosis [11,18]. Consequently, macrophages, which play a critical role in the body’s response to foreign bodies, recognize opsonized MNPs and internalize them through endocytosis, facilitating their removal, with the liver and spleen showing the highest uptake [19]. Thus, as the scaffold dissolves, chitosan minimizes the adverse effects and ensures the safe elimination of MNPs, promoting a more effective integration of the new tissue.

### 2.2. Biological Response

**Figure 9:**
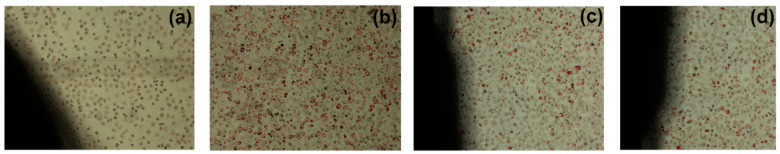
Inverted digital microscope images at 100× magnification of the L929 cell line of the areas of discoloration around the agar diffusion test scaffolds: (**a**) positive control (toxic latex), (**b**) negative control (quantitative filter paper), (**c**) S2, and (**d**) S4.

Figure 9 illustrates the cytotoxic behavior of the scaffolds.

In the cytotoxicity evaluation of the scaffolds, the analysis of the decoloration index was performed by calculating the average dimensions of the halo observed around each sample in four quadrants. The positive control (Figure 9a) exhibited a severe degree of cytotoxicity, classified as grade 4, with a decoloration zone of 0.888 cm. This result confirms that the positive control is highly cytotoxic, as expected. In contrast, the negative control (Figure 9b) showed no cytotoxicity, with a grade of 0 and a decoloration zone of 0.0 cm. The magnetic scaffolds analyzed, S2 (Figure 9c) and S4 (Figure 9d), indicated no cell lysis, classifying them as non-cytotoxic materials, both with a grade of 0 and a decoloration zone of 0.0 cm.

These results suggest that similar to the negative control, these samples do not exhibit cytotoxic properties. This finding highlights the absence of toxicity in these structures towards cells, reinforcing their potential safe applicability in biomedical contexts. The non-toxic behavior of the scaffolds was expected, as previous research [61,62] indicates that cobalt ferrite demonstrates non-toxic behavior at low concentrations, and its interaction with chitosan enhances the cellular viability of the system.

**Figure 10:**
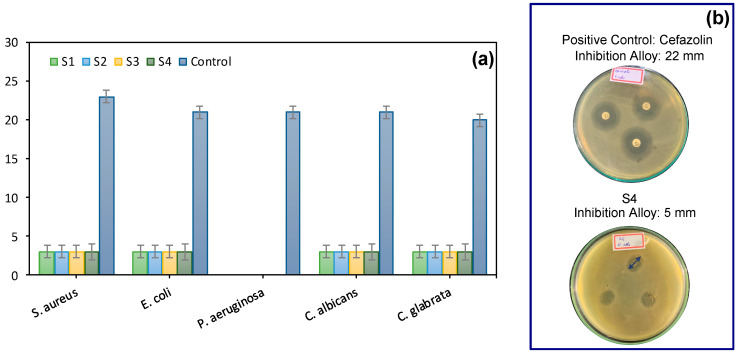
Antimicrobial activity: (**a**) zone of inhibition of the scaffolds against *S. aureus*, *E. coli*, *P. aeruginosa*, *C. albicans*, and *C. glabrata*. The error bars indicate the mean ± SD (*n* = 3), representing the significant differences between S1, S2, S3, S4, and the control. (**b**) Cefalozin (positive control) and S3 scaffold inhibition zone images of representative disk diffusion test plates.

Figure 10a,b illustrates the antimicrobial activity of the scaffolds.

The antibacterial activity of the scaffolds was determined by the modified agar disc diffusion method against *S. aureus.*, *E. coli*, *P. aeruginosa*, *C. albicans*, and *C. Glabrata* (Figure 10a). The antimicrobial efficacy was visually determined by measuring the inhibition zone formed around the discs, allowing the classification of inhibition as high (above 20 mm), moderate (10 to 20 mm), or low (below 10 mm) [63]. The results, including the diameters of the inhibition zones for each tested microorganism, are presented in Figure 10. Figure 10b illustrates the formation of inhibition zones resulting from microorganisms with which effective antimicrobial activity was obtained.

The analysis of data from Figure 10a reveals that the scaffolds (S1, S2, S3, S4) demonstrated consistent antimicrobial activity against nearly all tested microorganisms (*S. aureus*, *E. coli*, *P. aeruginosa*, and *C. albicans* e *C. glabrata*), with a uniform colony-forming unit (CFU) count of 5. A comparison of the scaffold results with the control standard, which showed a CFU count of 20 to 23, revealed notable antimicrobial efficacy against *S. aureus*, *E. coli*, *C. albicans*, and *C. glabrata*. This efficacy was achieved without the use of drugs or components with specific microbial activity, suggesting that the scaffolds possess intrinsic antimicrobial properties. This result is particularly significant for biomedical applications, where infection prevention is critical. However, a reduced efficacy was observed against *Pseudomonas aeruginosa*, indicating a limitation in the antimicrobial activity of the scaffolds against this microorganism.

The scaffolds’ antimicrobial capabilities are crucial for effectively protecting injured areas against infections, thereby promoting faster healing and more efficient tissue regeneration [64]. These properties were one of the main reasons for selecting the S3 sample for additional time kill assay and minimum inhibitory concentration (MIC) tests in order to prove its antimicrobial behavior. Sample S3 exhibited an optimized profile in terms of porosity, hydrophilicity, and compressive strength while showing superior bactericidal potential. In contrast, sample S1 was chosen as a reference due to its lower performance, allowing for a clear comparison of the bactericidal test results. These tests aim to explore S3’s bacterial elimination capabilities further, reinforcing its potential application in the biomedical field.

**Figure 11:**
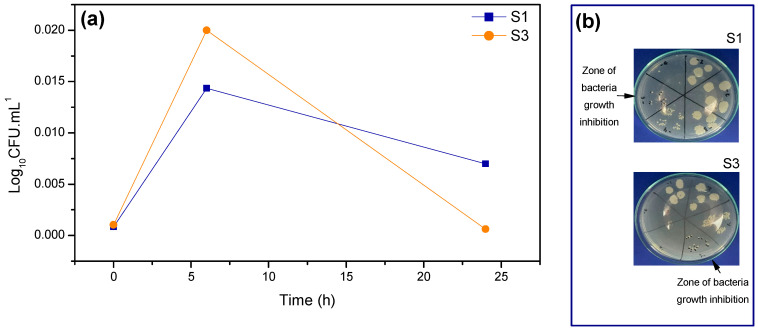
Time kill curve. (**a**) Scatterplot with straight lines and markers of *Staphylococcus aureus* inhibition test results for S1 and S3. (**b**) S1 and S3 zones of bacterial growth inhibition images.

Figure 11 illustrates the time kill curve of *S. aureus* when in contact with scaffolds S1 and S3.

In Figure 11, the time kill curve of *Staphylococcus aureus* is illustrated over time. The exponential growth phase, where microbial cells reproduce and increase exponentially in number, is represented in Figure 11a between 0–6 h. The decline/death phase, characterized by a logarithmic decrease as cell deaths exceed the number of new cells, occurs between 6–24 h. A similar behavior was observed in a study that developed a multifunctional nanofibrous scaffold made of polyvinyl alcohol (PVA), sodium alginate (SA), and silk fibroin (SF) loaded with asiaticoside (AT) to treat diabetic ulcers in rats, where the AT-loaded nanofibrous structures were more effective in causing cell death during the 6–24 h period [64].

Although S1 exhibited a lower exponential growth phase compared to S3, the decline phase of S1 does not show a consistent downward trend, indicating likely bacteriostatic activity. On the other hand, S3 displays a constant logarithmic decline, suggesting that from 6 to 24 h, the CFU count is approximately zero, demonstrating potential bactericidal activity and synergy in the antibacterial performance of the magnetic S3 scaffold. Notably, the efficacy of S3 in eradicating *S. aureus* progressively increased, reaching its peak antibacterial activity after 24 h, underscoring its effectiveness.

Table 4 details the MIC (minimum inhibitory concentration) and MBC (minimum bactericidal concentration) values of the cobalt ferrite particles synthesized by combustion reaction and scaffolds S1 and S3.

The results presented in Table 4 indicate the antimicrobial efficacy of cobalt ferrite particles and scaffolds S1 and S3 against *Staphylococcus aureus* MRSA and *Escherichia coli*. The CFO particles exhibited significant antimicrobial activity, with a MIC of 6.25 μg/mL against *S. aureus* MRSA. Given that the *S. aureus* strain is resistant to common antibiotics such as methicillin, this highlights the potential of cobalt ferrite particles being used as effective antimicrobial agents for combating resistant bacterial infections. For *E. coli*, the MIC was 12.5 μg/mL, indicating moderate efficacy according to Gole et al. [65].

Scaffold S1 exhibited a MIC of 7.5 μg/mL for both *S. aureus* and *E. coli*, suggesting that chitosan also has significant antimicrobial properties against Gram-positive and Gram-negative bacteria. On the other hand, scaffold S3 had a MIC of 37.5 μg/mL for both bacteria, indicating that the incorporation of 7.5% CFO enhances antimicrobial efficacy.

When comparing these results with those from other studies, such as Gole et al. (2020) [65], which reported a MIC of 25 µg/mL for cobalt ferrite particles synthesized by co-precipitation against *S. aureus*, it is evident that CFO synthesized by the combustion method demonstrates superior antimicrobial activity against the resistant strain (*S. aureus* MRSA). Additionally, compared to studies by Gingasu et al. and El-Shahawy et al. [66,67], which observed effective inhibition against *S. aureus* and *E. coli* using chitosan–cobalt ferrite nanohybrids, the results for S1 and S3 indicate a synergistic effect of cobalt ferrite combined with chitosan.

The bactericidal action observed in scaffold S3 follows a multi-stage mechanism, as reported by El-Shahawy et al. [67]. Initially, chitosan facilitates the adhesion of CFO nanoparticles to the bacterial cell membrane, disrupting essential functions such as permeability and cellular respiration. Subsequently, these nanoparticles penetrate the bacterial cell, interacting with sulfur and phosphorus compounds, causing internal damage. Inside the bacterium, the nanoparticles can interact directly with DNA, inhibiting its replication and ultimately leading to cell death. Additionally, cobalt ferrite nanoparticles release cobalt ions, which contribute further to the antibacterial activity, enhancing the overall effect of the material on the bacteria. This finding underscores the potential of sample S3 as an effective and promising antimicrobial agent in biomedical applications.

## 3. Experimental Section

### 3.1. Materials

The ferric nitrate nonahydrate [Fe(NO_3_)_3_·9H₂O] PA-ACS was from Synth^®^, cobalt(II) nitrate hexahydrate [Co(NO₃)₂·6H₂O] PA-ACS was from Synth^®^, and the urea [CO(NH_2_)_2_] 99% was from Synth^®^. The chitosan (CS) came from Sigma Aldrich^®^, Merck Group (Darmstadt, Germany), with a degree of deacetylation ≥90% and an average molecular weight of between 50–150 kDa; glacial acetic acid (PA) came from Vetec^®^ Química Fina. (Rio de Janeiro, Brazil); Ammonium hydroxide solution (NH_4_OH) with a purity of 99% was sourced from Neon^®^ (Suzano, Brazil), concentration of 28–30%; and the phosphate buffer solution (PBS), pH 7.4, came from Sigma Aldrich^®^, Merck Group (Darmstadt, Germany). All precursors were used as received without further processing or treatment.

### 3.2. Methods

#### 3.2.1. Synthesis of Cobalt Ferrite (CoFe_2_O_4_) via Combustion Reaction

For the synthesis of ferrite, cobalt nitrate (Co(NO_3_)_2_) was used as the oxidizing agent, and urea (CO(NH_2_)_2_) as the fuel and reducing agent. The initial composition of the solution was based on the total valence of the oxidizing and reducing agents, using concepts from propellant and explosive chemistry to achieve the stoichiometric oxidizer/fuel ratio, Φe = 1 [68], to establish the stoichiometry of the desired phase. The redox mixture of nitrates and fuel was subjected to direct heating in a stainless steel container attached to a conical reactor designed for combustion synthesis on a 200 g batch scale, which follows the theoretical balanced stoichiometric Equation (1) for cobalt ferrite:

2Fe(NO_3_)_3(aq)_⋅9H_2_O + Co(NO_3_)_2(aq)_⋅3H_2_O + 4CO(NH_2_)_2(aq)_⋅→ CoFe_2_O_4↓_ + 4CO_2↑_ + 29H_2_O_↑_ + 8N_2↑_ (1)

**Figure 12:**
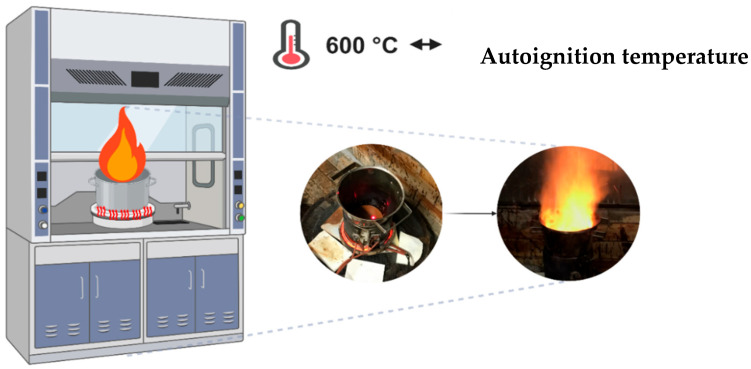
Cobalt ferrite combustion reaction on a pilot scale.

Figure 12 illustrates the synthesis of cobalt ferrite via the combustion reaction on a pilot scale.

#### 3.2.2. Scaffolds Preparation

The scaffolds were prepared by solution mixing as described in the patent device INPI: BR 10 2023 009515-1 [69]. First, solutions of (1.5% *w*/*v*) chitosan (1% acetic acid) were developed in a Nova Ética model M 110-VER-4K3 mechanical shaker (Vargem Grande do Sul, São Paulo, Brazil) for one hour at room temperature. Then, the CFO was added in the appropriate proportion, and the solutions were mixed for another hour. These steps were carried out under constant stirring at 346 rpm. The final solutions were poured into (20 mL) Petri dishes. The proportions of CFO/chitosan constituting the scaffolds are listed in Table 5 and were determined according to the method by Farzaneh et al. [42]

**Figure 13:**
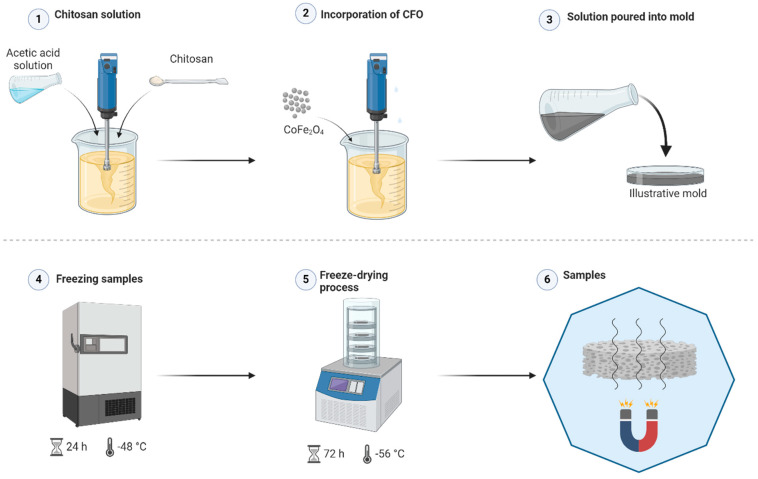
Scaffold fabrication process.

The molds were kept at −48 °C/24 h for freezing in an ultra freezer (AmericanLab—490L). The frozen scaffolds were freeze-dried to obtain a 3D porous structure in a stainless steel L108 freeze-dryer (AISI304-LIOTOP) at −56 °C/72 h. The scaffolds were carefully adjusted to pH 7 using a (1 M) NH_4_OH atmosphere under a glass dome for 24 h. After this period, the scaffolds were kept at −48 °C/4 h and lyophilized again for 24 h. Figure 13 illustrates the scaffold processing flowchart.

### 3.3. Physicochemical Characterization

The Fourier transform-infrared spectroscopy (FT-IR) technique was used to identify the functional groups in the scaffolds, employing a Bruker spectrometer model. Measurements were conducted over the 4000 to 400 cm^−1^ range with a resolution of 4 cm^−1^. X-ray diffraction (XRD) analysis was performed using a Bruker D2 PHASER instrument with Cu Kα radiation, operating at 30 kV and 10 mA, scanning 2θ angles from 10° to 60° in 2° increments at a rate of 2°/min. The crystallite size was calculated with the aid of the Scherrer Equation [70] and from the peak of the most intense basal reflection. Morphological analyses (SEM) were carried out using a scanning electron microscope (Tescan VEGA3 SBH) (Brno, Czech Republic) equipped with EDS (Oxford Instruments—model X-ACT IE15). The microscope operates at a current between 10 and 15 mA and an accelerating voltage of 10 kV, with a magnification of 5000× at room temperature. Four samples were analyzed in quintuplicate to ensure reproducibility. Mercury porosimetry measurements were conducted using a Micromeritics Autopore IV porosimeter. The magnetic properties were evaluated using a vibrating sample magnetometer (MicroSense EZ9) with a maximum magnetic field of 13.700 G at room temperature. The swelling potential (SP) was assessed in PBS (pH 7.4) for 24 h with three replicates. The degree of swelling was calculated using Equation (2):

(2) $$SD\left(\%\right)=\frac{W-W_{0}}{W_{0}}\times 100\%$$

where W_0_ is the initial sample weight, and W is the final sample weight. The contact angle was measured with an AGC-002 digital kinetic goniometer using drops of deionized water, and images were taken after 1 s. The compression tests were performed according to ASTM D 1621-00 using cylindrical specimens and an INSTRON 3366 universal testing machine. The compressive strength was calculated using Equation (3):

(3) 4Fσ=πD2

where F is the maximum load applied (N), and D is the diameter of the test specimen (mm).

The test was conducted based on the ASTM F1635-1 standard to evaluate the scaffold degradation during immersion and exposure in PBS solution. The samples were divided into two experimental groups with a diameter and thickness of 1 cm². Five samples from each group were initially weighed using a precision analytical balance, immersed in 7.5 mL of PBS solution, covered with PVC film, and incubated in a bacteriological oven at 37 °C for 14 and 21 days. The weight loss (WL) was calculated using Equation (4), which determines the percentage difference in the sample masses before and after the degradation test.

(4) $$WL\left(\%\right)=\frac{W-W_{0}}{W_{0}}\times 100\%$$

where W_0_ is the initial sample weight, and W is the final mass of the sample (after degradation).

### 3.4. Biological Analyses

Cytotoxic activity was evaluated by the agar diffusion method, according to the ISO 10993-5:2009 standard [71], using L929 mouse fibroblast cells (ATCC NCTC clone 929, Cell Bank of Rio de Janeiro, Brazil). The cells were cultured in the RPMI 1640 medium (Gibco—Invitrogen Corporation, Grand Island, NE, USA) and incubated at 37 °C with 5% CO_2_ until reaching 80% confluence. Then, trypsinization was performed with 0.25% trypsin (Gibco^®^, Life Technologies), and cell counting was conducted using an automated cell counter from Interwoven—Thermo Fisher (Waltham, MA, USA). Suspensions of 1.0 × 10^5^ cells/mL were distributed into 6-well plates, with 4 mL added to each well and incubated under the same conditions for 24 h. Then, 2× concentrated MEM medium (Gibco^®^—Invitrogen Corporation, Grand Island, NE, USA) and agar solution with neutral red (Sigma-Aldrich, St. Louis, MO, USA) were added. Scaffolds, as well as the latex and filter paper, were sectioned into 1 cm^2^ pieces and inserted into the wells, with a positive control (toxic latex sheet) and a negative control (quantitative filter paper with a pore size of 0.45 µm, obtained from Química Moderna). The plates were incubated for 24 h, and the clear zones were quantified (ISO 10993-5). Cell lysis was evaluated using a NIKON ECLIPSE TS100 inverted digital microscope (Minato, Tokyo, Japan).

For antimicrobial activity, the following standard strains from the American Type Culture Collection (ATCC) were utilized for testing: Methicillin-resistant *Staphylococcus aureus* (MRSA, ATCC:43300), *Staphylococcus aureus* (ATCC:77193), *Escherichia coli* (ATCC:25922), *Pseudomonas aeruginosa* (ATCC:27853), *Candida albicans* (ATCC:14053), and *Candida glabrata* (ATCC:90030).

The agar diffusion method used strains of *S. aureus*, *E. coli*, *P. aeruginosa*, *C. glabrata*, and *C. albicans*. The inoculum was prepared using tryptic soy broth (TSB) for the bacteria and Sabouraud dextrose broth for the fungi. Each bacterial and fungal strain loop was suspended in 5 mL of these broths and incubated in a growth chamber for 24 h at 35 °C for the bacteria and 48 h at 25 °C for the fungi. After incubation, the microorganisms were standardized by spectrophotometry in the visible range at 580 nm with 25% transmittance, corresponding to a concentration of 1 × 10^8^ CFU/mL (colony-forming units per milliliter). Finally, a 50 µL aliquot (1%) of the standardized inoculum was added to 4.95 mL of Mueller–Hinton agar for the bacteria and Sabouraud dextrose agar for the fungi. A double-layered agar was used in the method. Sterile Mueller–Hinton agar plates for the bacteria and Sabouraud dextrose agar plates for the fungi were used as the bottom layer in a quantity of 2 mL. The top layer (5 mL) containing 1% standardized inoculum (*v*/*v*) was added. After the agar solidified, 8 mm diameter scaffolds and 8 mm diameter stainless steel templates for antimicrobial control were applied with 100 µL of the antimicrobials on the templates. All scaffolds were run in triplicate. The plates were incubated at 35 °C for 24 h for the bacteria and at 25 °C for 48 h for the fungi. Readings were taken using an inhibition zone reader and expressed in mm. Cefazolin (Gram-positive), gentamicin (Gram-negative), and nystatin (fungi) were used as the antimicrobial controls, all at a concentration of 200 µg/mL [72].

The time kill assay, a method that allows examining the kinetics of bacterial death when exposed to the test sample, was conducted strictly following the Clinical and Laboratory Standards Institute (CLSI) guidelines [73]. This approach ensures the methodological rigor and reproducibility of the results [74,75]. The assay included scaffolds (5 × 1 cm strips), a negative control, and a positive control using ciprofloxacin at 1.0 μg/mL for *Staphylococcus aureus* (ATCC:77193) [64]. In separate test tubes, each sample was incubated in 1 mL of bacterial culture (adjusted to 1 × 10^6^, exceeding the CFU/mL as the initial inoculum). The positive control tubes contained antibiotics at sub-MIC concentrations. The tubes were incubated for 24 h at 37 °C in a bacteriological incubator. Colony-forming units (CFU/mL) were determined at 0, 6, and 24 h by plating (100 µL) of the appropriate dilution factor onto nutrient agar plates. The plates were incubated at 37 °C, and the CFUs were counted after 24 h. Each experiment was performed in triplicate to ensure the reliability of the data. After incubation, CFU/mL were counted for each sample according to Equation (5):

(5) $$\frac{UFC}{mL}=\frac{\left(Average\;colony\;count\times Dilution\times 1000\right)}{Drop\;volume\;in\;µL}$$

The time kill curve was constructed by plotting the logarithm of colony-forming units per mL on the vertical axis against time on the horizontal axis. The bactericidal activity was evidenced by a reduction of 99.9% (≥3Log_10_) in the total colony-forming units per mL in the initial sample. The bacteriostatic activity was demonstrated by maintaining the original inoculum concentration or by a decrease of less than 99.9% (˂3Log_10_) in the total number of colony-forming units per mL in the initial sample.

The microbial sensitivity of the tested samples was determined following the protocol by Ellof et al. [76] using the broth microdilution method. The minimum inhibitory concentrations (MICs) were determined in 96-well microdilution plates according to the Clinical and Laboratory Standards Institute M07 and M27 protocols [77]. In each well of sterile, round-bottom plates, 190 μL of TSB broth was added, followed by 10% (*w*/*v*) CFO powder and 1.5% or 7.5% (*w*/*v*) of scaffolds S1 and S2, respectively. Serial dilutions of the CFO were performed to achieve final concentrations ranging from 100 × 10^3^ μg/mL to 390.62 μg/mL. Subsequently, 10 μL of a bacterial suspension (1.5 × 10^8^ CFU/mL) was added to each well, and the plates were incubated at 35 ± 2 °C for 24 h. The positive control included antibiotics (cephalexin hydrochloride at 200 μg/mL), and the negative control was performed with a culture medium without bacteria. After incubation, 20 μL of 2% TTC was added to each well, followed by an additional hour of incubation for visual result reading. The minimum bactericidal concentration (MBC) was performed for all wells showing inhibition in the microplate test. For each well, the material was transferred to Petri dishes containing Mueller–Hinton agar using sterilized sticks. These plates were incubated at 37 °C ± 1 for 24 h. The MBC was defined as the lowest concentration that showed no microbial growth. The reading was performed visually after the necessary incubation time [78].

The characterization and biological assays were performed by calculating the mean and standard deviation for each sample to ensure the reliability of the data and the reproducibility of the results. The mean and standard deviation were calculated from five replicates for the physical characterization tests (swelling behavior, contact angle, biodegradation, and mechanical strength) and from three replicates for the antimicrobial activity assays.

## 4. Conclusions

In summary, chitosan scaffolds that are magnetically activated with cobalt nano ferrite and synthesized using a pilot-scale combustion reaction were obtained sustainably and were non-toxic. Increasing the concentration of the nanoparticles thus directly influenced the magnetization, compression modulus, swelling behavior, biodegradation, and porosity of the scaffolds. An optimum behavior was observed with a concentration of 7.5% CFO, resulting in a mechanical improvement with a compressive strength of 87 kPa. In addition, the cytocompatibility of the scaffolds showed that they are safe to use for biomedical purposes and offer bactericidal and bacteriostatic activities. Future studies involving cell tests and in vivo trials could provide a greater understanding of the interaction of scaffolds with biological tissues, broadening their spectrum of clinical applications.

## Figures and Tables

**Table 1:** Main characteristic vibrations of the scaffolds compared with the literature.

Observed Frequency (cm^−1^)	Absorption Band	Assignment	Frequency Reported in the Literature (cm^−1^)	Reference
~432	Fe-O stretch	Axial Stretching of Fe–O in Octahedral Sites	451	[36]
~518	Fe-O stretch	Axial Stretching of Fe-O in Tetrahedral Sites	562	
~1021	C–O–C stretch	Axial Stretching of C–O–C	1065	[37]
~1367	C–N stretch	Axial Stretching of C-N in Primary Amines	1386	
~1570	N–H bend	Angular Deformation of N-H in Amide II	1590	[38]

**Table 2:** Porosity and average pore diameter of scaffolds S1, S2, S3, and S4.

Sample	Porosity (%)	Average Pore Diameter (µm)
S1 *	78	104.9
S2	79.5	131.3
S3	80	120.5
S4	83	87

* Reference specimen.

**Table 3:** Ms, Mr, and Hc of CFO and scaffolds S1, S2, S3, and S4.

Sample	Ms (emu/g)	Mr (emu/g)	Hc (Oe)
S1 *	0.16	0.002	265
S2	3.3	1.5	1095
S3	4.8	2.2	1274
S4	4.3	2.0	1275

* Reference specimen.

**Table 4:** MIC and MBC values. The values reported represent the mean MIC and MBC values obtained in triplicate against *S. aureus* MRSA and *E. coli* bacteria.

Samples	Microrganisms MIC/MBC (μg.mL^−1^)			
	*S. aureus* MRSA ATCC (43300)		*E. coli* ATCC (25922)	
	MIC	MBC	CIM	CBM
CFO	6.250 ± 0	-	12.500 ± 0	12.500 ± 0
S1	7.500 ± 0	-	7.500 ± 0	7.500 ± 0
S3	37.500 ± 0	-	37.500 ± 0	37.500 ± 0
Cefalexin	-	-	3.125 ± 0	50.000 ± 0

**Table 5:** Composition of scaffolds with a matrix of chitosan (CS) and a filler of CoFe_2_O_4_ (CFO).

Sample	Cobalt Ferrite (CFO)	Chitosan (CS)
S1 *	0%	100%
S2	5%	95%
S3	7.5%	92.5%
S4	10%	90%

* Reference specimen.

## Data Availability

Data are contained within the article.
